# Different Effects of Endothelial Extracellular Vesicles and LPS-Induced Endothelial Extracellular Vesicles on Vascular Smooth Muscle Cells: Role of Curcumin and Its Derivatives

**DOI:** 10.3389/fcvm.2021.649352

**Published:** 2021-05-31

**Authors:** Debiao Xiang, Yamei Li, Yuling Cao, Ying Huang, Lili Zhou, Xiulian Lin, Yong Qiao, Xin Li, Duanfang Liao

**Affiliations:** ^1^The Third Hospital of Changsha, Changsha, China; ^2^Key Laboratory for Quality Evaluation of Bulk Herbs of Hunan Province, Hunan University of Chinese Medicine, Changsha, China; ^3^The First Affiliated Hospital of Hunan University of Chinese Medicine, Changsha, China

**Keywords:** atherosclerosis, endothelial EVs, vascular smooth muscle cell, inflammation, curcumin

## Abstract

**Background:** During the progression of atherosclerosis (AS), the vascular endothelial and smooth muscle cells are reciprocally regulated by extracellular vesicles (EVs). EVs have different effects on pathological and physiological processes due to the different cargoes contained in EVs.

**Purpose:** To study the effects of endothelial cells-derived EVs on normal and inflammatory conditions. To investigate the effects of curcumin and curcumin derivatives (Nicotinic-curcumin) on endothelial EVs.

**Methods:** EVs were isolated from human umbilical vein endothelial cells (HUVECs) by ultracentrifugation. To examined the effect of normal and LPS-induced endothelial cells-derived EVs on the proliferation of human aortic smooth muscle cells (HASMCs), the CCK-8 assay was performed. Transwell and wound healing assays were conducted to assess cell migration. The effects of EVs on lipid accumulation following treatment with oxidized low-density lipoprotein (Ox-LDL) were evaluated with the oil red O staining assay and HPLC. The number of EVs was calculated using the nanoparticle tracking analysis (NTA) and BCA. The expression levels of Rab27a and Rab27b that regulate the EVs secretion were measured by Western blotting assay. The differential expression of miRNAs in endothelial EVs and LPS-induced endothelial EVs was analyzed using miRNA-Sequencing (miRNA-Seq) and RT-PCR.

**Results:** Treatment with endothelial EVs reduced the proliferation and migration of HASMCs as well as lipid accumulation in HASMCs. However, treatment with LPS-induced endothelial EVs did not inhibit the migration of HASMCs or lipid accumulation, instead it promoted the proliferation of HASMCs. Treatment with the two types of EVs induced differential expression of several miRNAs, including miR-92a-3p, miR-126-5p, miR-125a-3p, miR-143-3p, etc. Moreover, 1 μg/mL LPS induction greatly increased secretion of endothelial EVs. Treatment with curcumin and nicotinic-curcumin reduced endothelial EVs secretion, possibly by inhibiting inflammation.

**Conclusion:** Endothelial EVs may confer beneficial effects on atherosclerosis by regulating vascular smooth muscle cell (VSMCs), whereas pro-inflammatory factors may disrupt this effect.

## Introduction

Atherosclerosis (AS) is a progressive disease characterized by abnormal accumulation of lipids and immune cells in the vascular wall. AS has been implicated in the development of several cardiovascular and cerebrovascular diseases such as myocardial infarction, angina pectoris, heart failure and stroke (1, 2). Meanwhile, AS is caused by diabetes, hypertension, dyslipidemia, and inflammation (3, 4). Atherosclerotic lesions are focal, asymmetrical thickening of the arterial intima, consisting of numerous cell types, connective tissue components and lipids (5). Vascular endothelial and smooth muscle cells as well as macrophages participate in the formation of atherosclerotic plaques (6). Several studies suggest that endothelial dysfunction, which occurs at the initial stage of atherosclerosis, is involved in the formation, progression and occurrence of complications related to atherosclerotic plaques (7, 8). Inflammation is an important marker for endothelial dysfunction. Specifically, several pro-inflammatory factors in serum such as oxidized low-density lipoprotein (ox-LDL), tumor necrosis factor-α (TNF-α), interleukin-1 (IL-1β) etc, activates endothelial cells (9, 10) to release several cytokines, which recruit circulating monocytes and leukocytes and as well-induces abnormal proliferation and migration of vascular smooth muscle cells. Vascular smooth muscle cells are one of the main components of the fibrous cap of atherosclerotic plaques. Inflammation increases permeability of vascular intima, allowing entry of large amount of lipids in to the arterial lumen (11). Vascular smooth muscle cells proliferate and migrate to the intima, ingest large amounts of lipids and transform into foam subtypes that ultimately form the atherosclerotic plaque.

Extracellular vesicles (EVs) are small (30 to 1,000 nm) bilayered structures secreted by numerous cells (12, 13). Given the numerous biomolecules in EVs including miRNAs, DNA and proteins, the vesicles are used as specific diagnostic biomarkers for several diseases (14–16). Numerous studies also show that EVs are potential therapeutic targets and drug delivery systems (17, 18). Almost all types of cells secrete EVs, which are abundantly present in stable form in various tissues and body fluids. EVs mediate communication between cells, and through their loaded molecules such as miRNA, proteins, and cytokines, they regulate functions of the target cells (12, 19).

The interaction between endothelial and vascular smooth muscle cells is essential in the development of cardiovascular disease. Intriguingly, EVs enhance the efficiency of such interactions. Particularly, EVs signals can regulate vascular tone of Smooth muscle cells (SMCs) (20). Excessive secretion of EVs in VSMCs under endoplasmic reticulum stress induces apoptosis of ECs and infiltration of inflammatory cytokines (21). MiRNAs are one of main molecules in EVs. They regulate post-transcription gene expression by binding to the 3′-untranslated region (UTR) of specific target mRNA sequences. This modulates protein expression by either promoting mRNA degradation or by blocking mRNA translation. miR-126-3p derived from endothelial EVs modulates proliferation of vascular smooth muscle cell (VSMCs) and disrupts neointima formation by inhibiting secretion of Low-density lipoprotein receptor-related protein 6 (LRP6) (22). Uptake of miR-143/145 containing EVs provides atheroprotection in smooth muscle cells. MiR-125a-3p inhibits proliferation and migration of VSMCs and the development of vascular stenosis by targeting MAPK1 (23). Over-expression of miR-155 promotes the formation of foam cells and aggravates atherosclerosis by targeting SOCS1 via STAT3 and NF-κB signaling pathway (24). It has been demonstrated that miRNA-10b regulates proliferation of VSMCs via the Akt pathway, specifically by targeting TIP30 (25). EV derived miRNAs are thought to participate in the development of cardiovascular disease. Functions RNAs have been extensively studied. Expression of miRNA in endothelial EVs under physiological and disease conditions suggests these structures play critical roles in such disease conditions. In this paper, we evaluated the effect of regulating the expression of miRNAs in endothelial derived EVs.

**Scheme 1:**
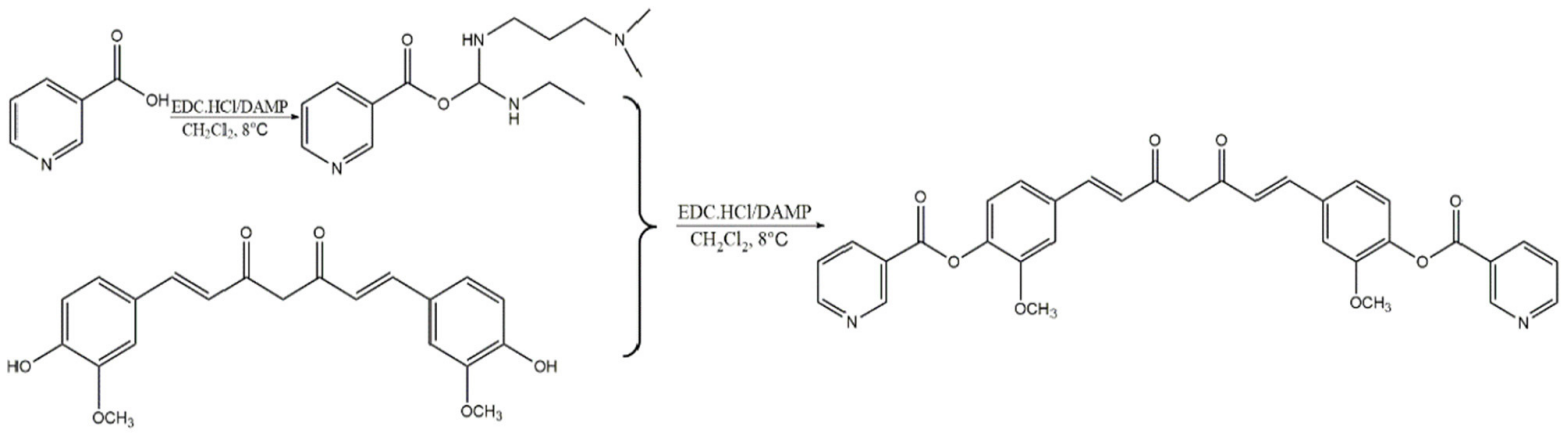
Synthesis of Nicotinic-curcumin (NC).

Curcumin isolated from the rhizome of *Curcuma longa* L. possess antioxidant, anti-inflammatory, antimicrobial, anticarcinogenic and cardiovascular protective properties (26). It has been evidenced that curcumin reduces the incidence of atherosclerosis due to its hypolipidemic effects, antioxidant and anti-inflammatory activity (27). Moreover, vast targets inevitably bring vast unnecessary side effects and low bioavailability of curcumin also restrict its clinical application. Nicotinic-curcumin (NC) is derivative of curcumin and niacin, synthesized through esterification (Scheme 1). We aimed to improve bioavailability of curcumin and combine the effects of curcumin with the lipid-lowering effects of niacin in atherosclerosis treatment. At present, we have found NC treatment remarkably increased efflux of cholesterol and reduced accumulation of ox-LDL in THP-1 cells (28).

## Materials and Methods

### Materials

Niacin and curcumin were purchased from Sinopharm Chemical Reagents Co., Ltd (Beijing, China). ECM medium and human aortic smooth muscle cells (HASMCs) were purchased from ScienCell Co., Ltd (San Diego, USA). ELISA kits were purchased from Multi Sciences Co., Ltd (Shanghai, China). Other materials included antibodies from Abcam (London, England), Oil red O stain from Solarbio Science & Technology Co., Ltd. (Beijing, China) and Cell culture flasks and transwell plates from Corning (New York, USA).

### Cell Culture

Human umbilical vein endothelial cells (HUVECs) were isolated from the human fresh umbilical cord based on the CD31 marker protein, using an immunofluorescence microscope They were cultured at 37°C in ECM medium (ScienCell, USA) in a humid incubator under 5% CO_2_ and 95% relative humidity (RH). Human aortic smooth muscle cells (HASMCs) were cultured in Dulbecco's modified Eagle medium (DMEM) (HyClone, Australia) supplemented with 10% fetal bovine serum (FBS), 1% of penicillin (100 U/mL) and streptomycin (100 μg/mL). The antibiotics were first diluted in dimethyl sulfoxide **(**DMSO) and culture medium to obtain the desired concentrations.

### Isolation and Identification of Endothelial EVs

HUVECs were cultured in Endothelial Cell Medium (ECM) (free of EVs). The supernatant was collected and centrifuged sequentially at 300 g for 10 min, 2,000 g for 10 min and 10,000 g for 30 min) under 4 °C. The supernatant was further centrifuged at 100,000 g for 70 min at 4 °C. The precipitate was resuspended in phosphate buffered solution (PBS) and centrifuged at 100,000 g for 70 min at 4 °C. Protein concentration in PBS (100~200μL) was quantified using the bicinchoninic acid (BCA) method. The EVs were stored at −80 °C.

### Uptake of Endothelial EVs by VSMCs

The cell culture medium was replaced with 2 mL of Exoperfect exo-CD63-GFP tracer virus medium, supplemented with 5 μg/mL polybrene. After co-incubation for 8 h, the cells were observed under the microscope before replacing the EV-free serum-based media. Incubation was continued for another 48 h before adding 2 μg/mL puromycin for screening CD63-GFP stably transfected cells. Normal cells served as controls. The culture medium was replaced every 2 days. Stable tranformants in the virus infected group served beyond death of all cells in the control group. The concentration of puromycin in the stable cells was halved in the subsequent culture. The transfected cells were cultured for 48 h in EVs-free medium and centrifuged to obtain the supernatant. The EVs were labeled using CD63-GFP before isolation using ultra-high centrifugation. VSMCs were cultured in medium containing CD63-GFP-labeled EVs for 12 h and 24 h. Cells were then fixed for 15 min using 4% paraformaldehyde before being sealed with DAPI tablets. The cells were observed and photographed using a laser confocal microscope.

### EVs Treatment and Co-culture of HUVECs and VSMCs

Purified endothelial EVs were diluted using DMEM medium and thereafter filtered using a bacterial membrane (0.22 μm) sieve. The VSMCs were then treated for 24 h using 20 μg/mL of endothelial EVs. A scratch was made in a monolayer of VSMCs co-cultured with HUVECs for 6 and 12 h to assess the migration ability of the VSMCs.

HUVECs were cultured for 8 h in the upper chamber of a 24-well-transwell plate (0.4 μm aperture) in ECM medium supplemented with 1 μg/mL of LPS, whereas HASMCs were cultured separately on a 24-well-plate. After activation using 1 μg/mL lipopolysaccharide (LPS), the HUVECs and HASMCs were co-cultured for 24 h before adding 2.5 nmol/mL GW4869 (inhibits EVs release) in an upper chamber of a 24-well-transwell plate. The proliferation of VSMCs in a lower chamber was assessing using the CCK-8 assay after 24 h culture. VSMCs in a lower chamber were reseeded onto upper chamber of separate transwell plates (8 μm aperture), incubated for 12 h before staining using 0.25% crystal violet. The stained cells were observed, photographed and counted under the inverted microscope.

### Determination of Free Cholesterol in Cells

The HASMCs were treated for 24 h with 80 μg/mL ox-LDL to established lipid accumulation model. The HASMCs were then centrifuged at 1,000 r/min for 10 min under 4°C, resuspended in 500 μL 0.9% NaCl solution and repeatedly (3 ~ 4 times) freeze-thawed at between −80°C and room temperature. The cell membranes of the HASMCs were ruptured using an ultrasonic instrument. The supernatant was centrifuged at 12,000 r/min for 10 min under 4°C, before the protein concentration in xyz was determined using the BCA method. To determine cholesterol concentration, equal volumes of the supernatant and KOH-ethanol were first mixed vigorously for 5 min to a uniform solution and saponified ultrasonically for 1 h in a water bath at 48°C. Thereafter, 6% trichloroacetic acid (45 μL) was added to denature the protein before adding Iso-0volumetric cholesterol extractor (n-butane: isopropanol at the ratio of 60:40). After mixing and centrifugation at 4°C (12,500 r/min, 10 min), the upper organic phase was collected, dried in nitrogen and dissolved in 40 μL acetonitrile. The concentration of free cholesterol was measured using HPLC (29). The aqueous phase was processed three times to ensure maximum cholesterol extraction.

## Results

### Identification of Endothelial EVs

**Figure 1:**
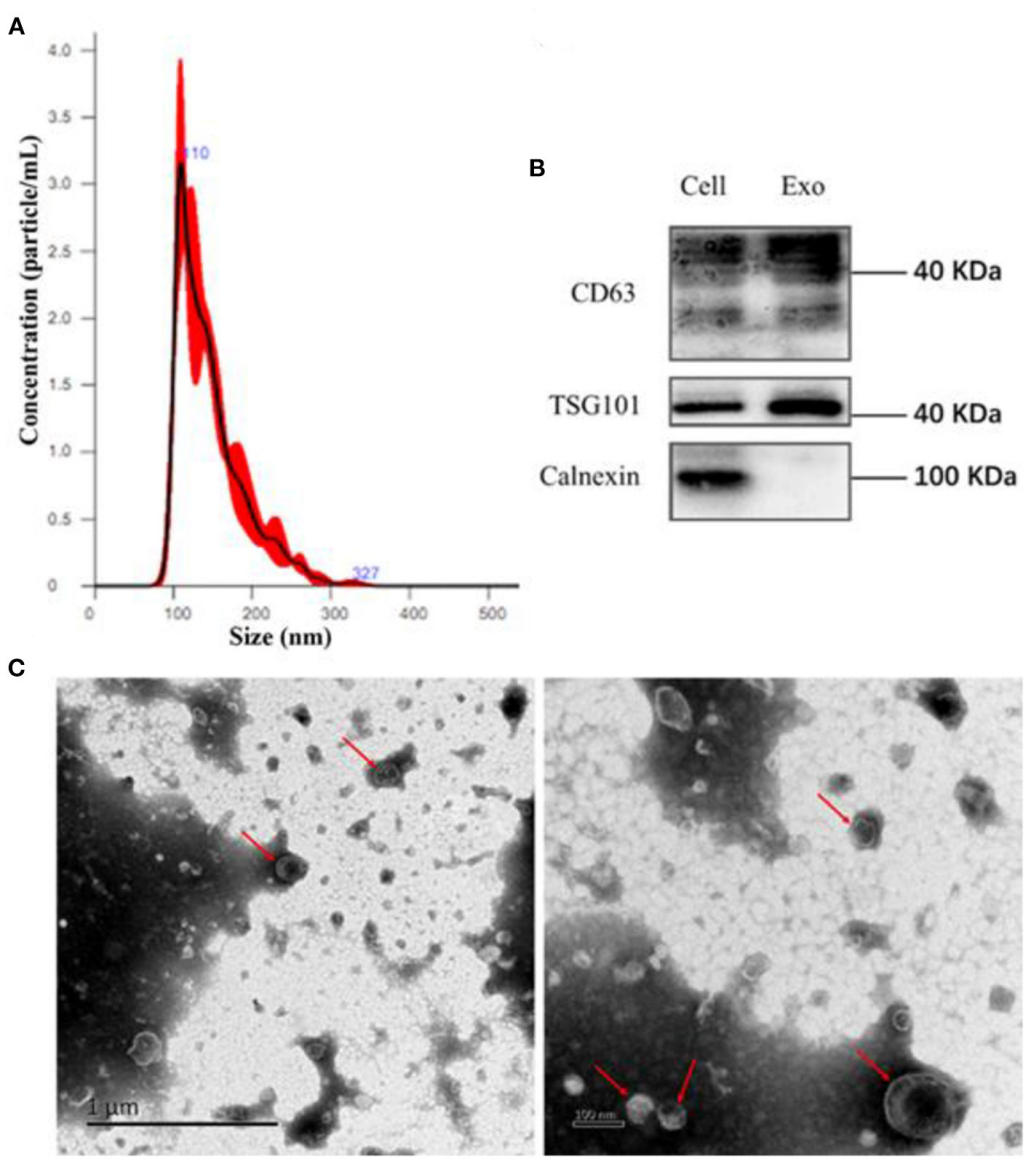
Isolation of EVs using ultra-high-speed centrifugation. **(A)** Determination of particulate size of EVs using nanoparticle Tracking Analysis NTA; **(B)** Western blot analysis for the expression of CD63, TSG101, and Calnexin on the endothelial EVs; **(C)** The morphology of EVs under a transmission electron microscope (TEM).

EVs are small bi-layer structures (30–1,000 nm) that carry numerous biomolecules such proteins, lipids, DNA and RNA. Information between cells is transmitted through the constituent proteins and nucleic acids. Endothelial cells secrete numerous biomolecules, and given their wide distribution, they interact with innumerable cells. Endothelial cells secrete cytokines, which are carried via EVs to downstream target cells such as vascular smooth muscle cells, macrophages, etc. Averagely, Endothelial EVs are 150 nm wide (Figure 1A). They contain CD63, TSG101, and Calnexin surface markers (Figure 1B). Transmission electron microscopy revealed that the endothelial EVs are generally spherical (Figure 1C).

### Effects of Endothelial EVs on the Proliferation, Migration, and Accumulation of Lipid in HASMCs

#### Uptake of Endothelial EVs by HASMCs

**Figure 2:**
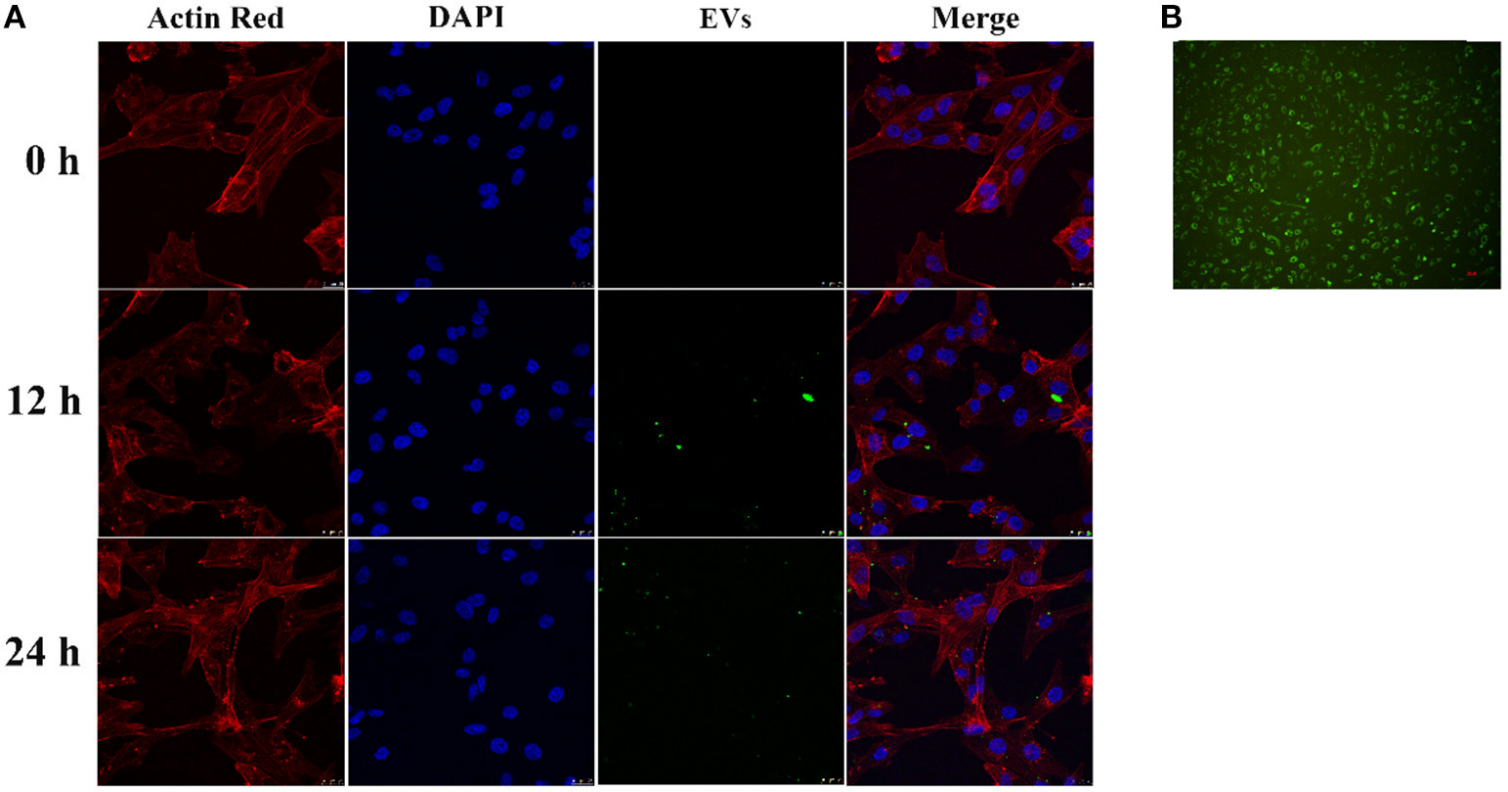
Uptake of CD63-GFP-EVs by vascular smooth muscle cells. **(A)** Transmission electron microscopy showing successful uptake of EVs by HASMCs; **(B)** Uptake of CD63-GFP by HUVECs.

CD63-GFP labeled EVs in culture supernatant of transduced HUVECs (Figure 2B) were isolated using an ultra-high centrifugation. Laser scanning confocal microscope revealed successful uptake of CD63-GFP-EVs by HASMCs after 12 h and 24 h of incubation (Figure 2A).

#### The Effects of Endothelial EVs on the Proliferation and Migration of HASMCs

**Figure 3:**
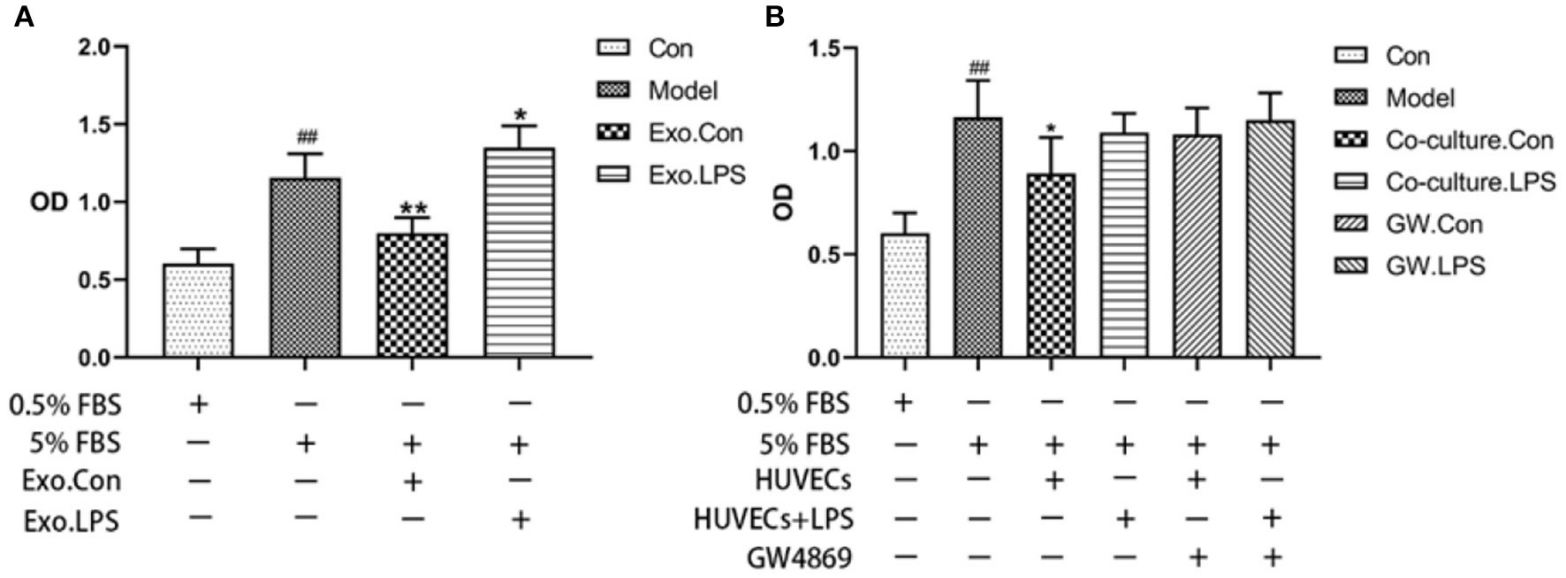
**(A)** The effect of EVs derived from LPS activated HUVECs (EV.LPS) on proliferation of HASNCS. The proliferation of HASMCs was assessed using CCK-8 assay; **(B)** Effect of HUVECs culture on proliferation of HASMCs in the presence of LPS. The assessment was performed using the CCK-8 assay (Data represents mean ± SD of 6 independent experiments, ^#^*p* < 0.05 and ^##^*p* < 0.01, respectively. *p < 0.05, and **p < 0.01, respectively). ^#^: Compared with Con, *: compared with Model.

Endothelial EVs substantially inhibited proliferation of HASMCs (*P* < 0.01). In contrast, EVs derived from LPS stimulated HUVECs (EV.LPS) promoted proliferation of HASMCs (*P* < 0.05; Figure 3A). In addition, LPS activated HUVECs exerted a similar effect on HASMCs (*P* < 0.05). However, culture with LPS stimulated HUVECs promoted the proliferation of HASMCs (Figure 3B).

**Figure 4:**
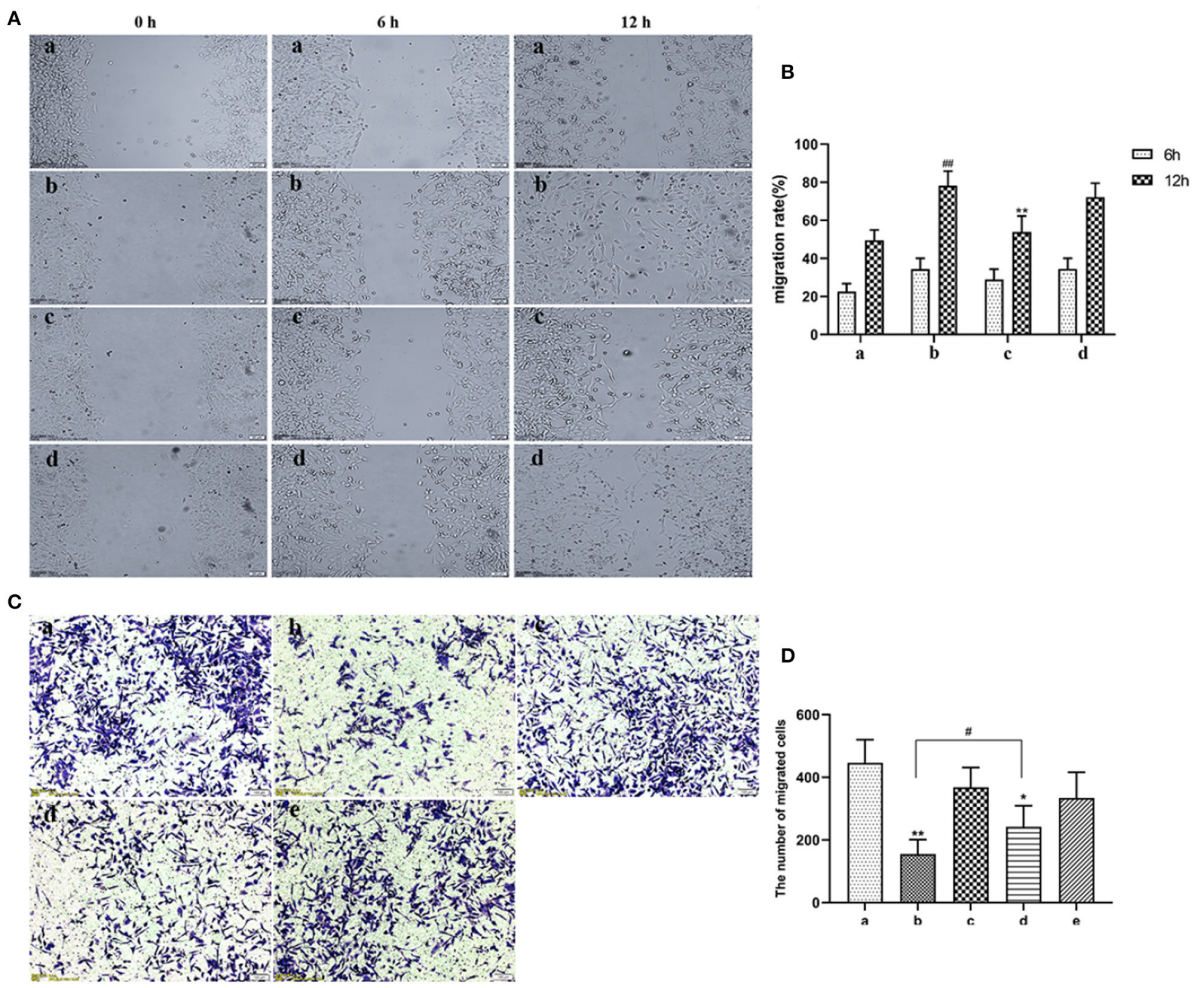
**(A)** Scratch wound assay for the effect of LPS on migration of HASMCs. (a) Control. (b) The experimental model. (c) Effect of EVs from LPS stimulated endothelial cells on migration of HASMCs. (d) Effect of EVs from LPS-stimulated endothelial cells (EV.LPS) on the migration of HASMCs. **(B)** The effect of LPS-activated HUVECs on migration of HASMCs; **(C)** Transwell assay for the effect of HUVECs, LPS and GW4869, either alone or in combination, on the migration of HASMCs (a) HUVECs culture alone. (b) HUVECs and HASMCs co-culture (Co-culture.Con). (c) HUVECs and HASMCs co-culture in the presence of 1 μg/mL LPS (Co-culture.LPS). (d) HUVECs and HASMCs co-cultured with 2.5 nmol/mL GW4869 (GW.Con). (e) HUVECs and HASMCs co-cultured in the presence of 2.5 nmol/mL GW4869 and 1 μg/mL LPS (GW.LPS). **(D)** Transwell assay for effect of GW4869 treatment on the inhibition property of endothelial EVs on the migration of HASMCs (Data represents mean ± SD of 6 independent experiments, ^#^*p* < 0.05 and ^##^*p* < 0.01, respectively. *p < 0.05 and **p < 0.01).

EVs secreted by non-activated HUVECs inhibited migration of HASMCs (*P* < 0.05). Wound scratch assay revealed that LPS-activated HUVECs had no effect on the migration of HASMCs (Figures 4A,B). Further analyses revealed that LPS activated HUVECs disrupted migration of HASMCs. A culture of non-activated HUVECs substantially modulated the migration of HASMCs (*P* < 0.05). However, GW4869 treatment inhibited the effect of endothelial EVs on proliferation and migration of HASMCs (Figures 4C,D).

#### The Effects of Endothelial EVs on Accumulation of Lipids in HASMCs

**Figure 5:**
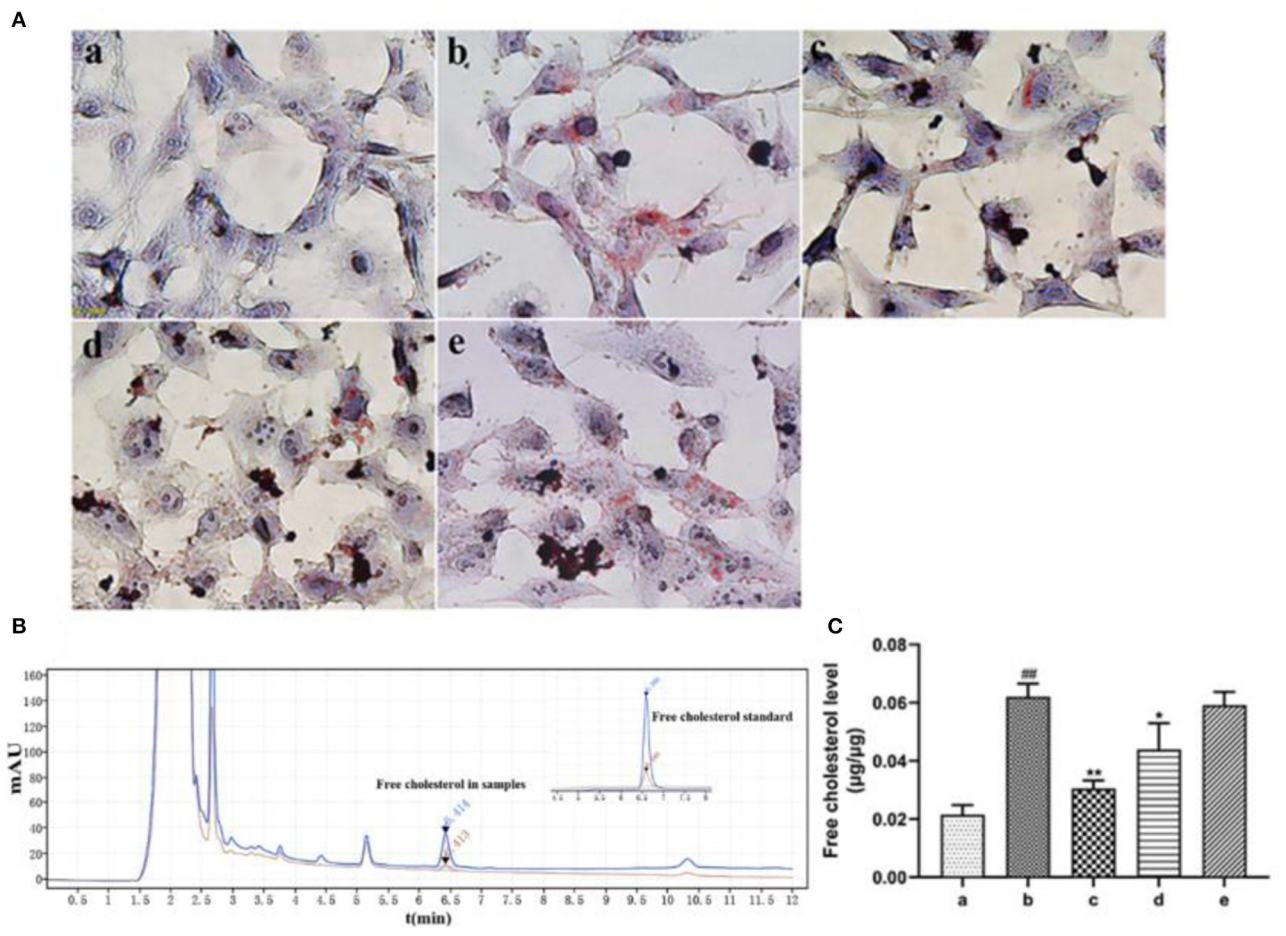
**(A)** Oil red O staining for the effect of ox-LDL treatment on lipid accumulation in HASMCs (magnification × 400) (a) Control. (b) Experimental model. HASMCs were treated with 80 μg/mL ox-LDL. (c) Inhibitory effect of rosuvastatin against augmented lipid accumulation in HASMCs mediated by ox-LDL. The cells received 1μM rosuvastatin treatment in the presence of 80 μg/mL ox-LDL. (d) Effect of EVs derived from non-activated HUVECs on lipid accumulation in HASMCs culture in the presence of 80 μg/mL ox-LDL. (e) Effect of EVs derived from LPS-activated HUVECs on lipid accumulation in HASMCs cultured in the presence of 80 μg/mL ox-LDL; **(B)** HPLC chromatography on retention time of free cholesterol in HASMCs. Measurement was performed at 205 nm (red) and 216 nm (blue). The retention time of free cholesterol in HASMCS was 6.415 min; **(C)** HPLC for the amount of lipids accumulated in HASMCs (Data represents mean ± SD of 6 independent experiments. ^#^*p* < 0.05 and ^##^*p* < 0.01, respectively. *p < 0.05 and **p < 0.01, respectively). ^#^: Compared with Con, *: compared with Model.

Oil red O staining revealed that ox-LDL treatment induced uptake of large amount of lipids by HASMCs. However, rosuvastatin treatment reversed this phenomenon (Figure 5A). EVs secreted by inactivated HUVECs (EV.con) inhibited accumulation of lipids in HASMCs, whereas EVs derived from LPS activated HUVECs (EV.LPS) had no effect on the aforementioned phenomenon. HPLC revealed the average retention time of free cholesterol in HASMCS was 6.415 min. Compared with EV.con (43.97 ± 9.02 ng/μg), ox-LDL treatment significantly enhanced retention of intracellular cholesterol (62.12 ± 4.49 ng/μg) (*P* < 0.05). Interestingly, EV.LPS treatment exerted comparable effect as ox-LDL (59.21 ± 4.55 ng/μg) (Figures 5B,C). These findings demonstrated that EVs from non-activated HUVECs (EV.con) reduces accumulation of lipids in HASMCs, in contrast to EV.LPS.

### LPS Disrupted the Expression of miRNAs in Endothelial EVs

**Figure 6:**
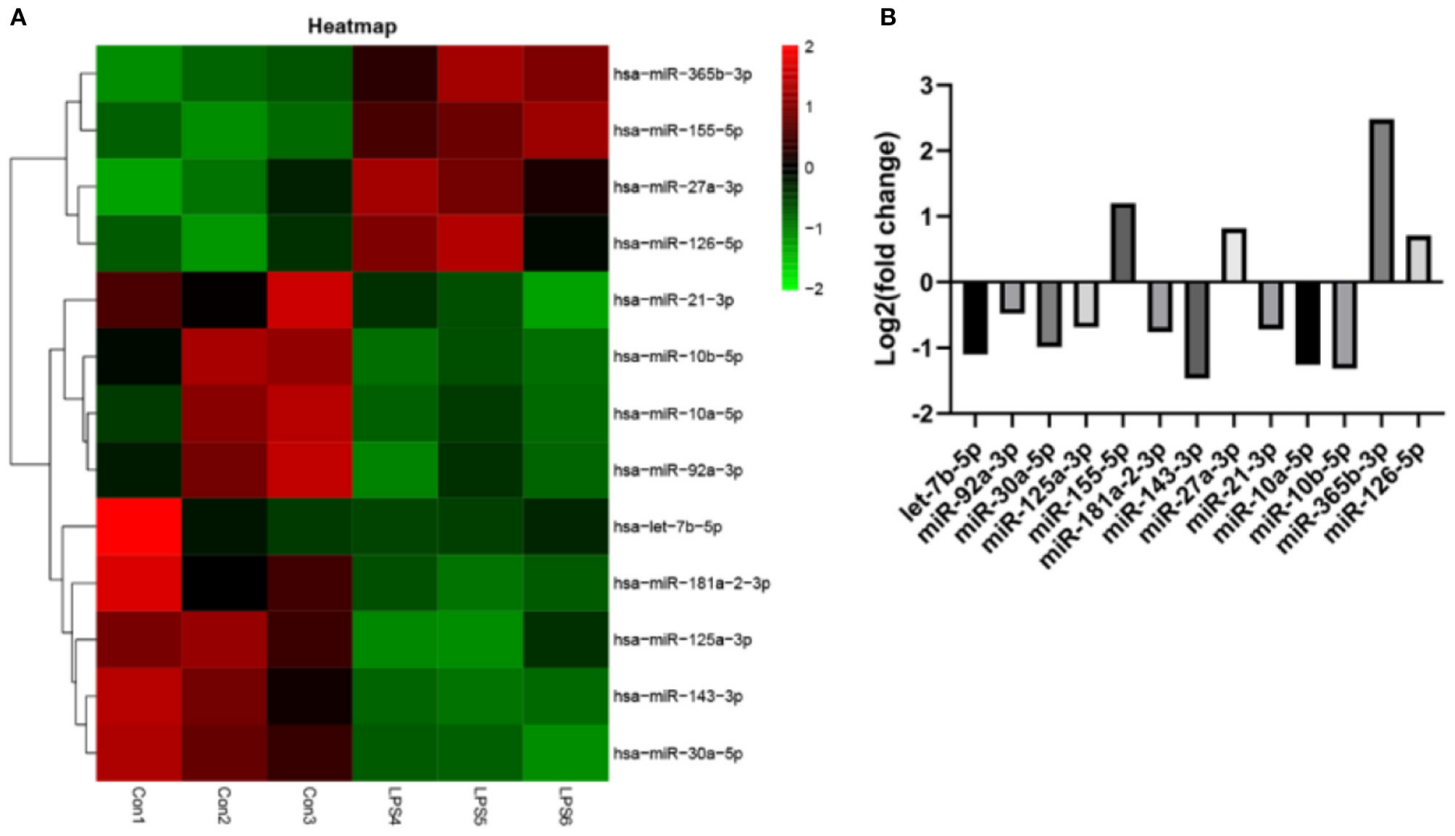
The effect of EVs from LPS activated HUVECS on the expression of miRNA. **(A)** Heatmap for microarray of the differently expressed miRNAs; **(B)** The top 13 most differently expressed miRNAs following EVs treatment, derived from LPS activated HUVECS.

EVs derived from non-activated HUVECs disrupted the relative expression of several miRNAs. Significant change in the expression of miRNAs was based on (fold change) >0 for up-regulated expression and log2 (fold change) <0 for down-regulated expression, both at *P* < 0.05. Particularly, LPS treatment dysregulated the expression of 234 miRNAs. Of these 124 were over-expressed whereas 110 were under expressed. Further assessment revealed that 13 of the most differently expressed miRNAs participated in various aspects of atherosclerosis. Of these, four miRNAs including miR-27a-3p, miR-365b-3p, miR-126-5p, and miR-155-5p were over-expressed. The rest, including let-7b-5p, miR-92a-3p, miR-30a-5p, miR-125a-3p, miR-181a-2-3p, miR-143-3p, miR-21-3p, miR-10a-5p, and miR-10b-5p (Figures 6A,B), were under-expressed.

### Curcumin and Nicotinic-Curcumin Inhibited LPS-Induced Endothelial Inflammatory Responses

Certain inflammatory cytokines can cause inflammation and disrupt the normal functioning of endothelial cells. In the early AS stage, inflammatory cytokines stimulate endothelial cells to produce adhesion molecules which recruit mononuclear macrophages as well as promote proliferation and migration of vascular smooth muscle cells. Mononuclear macrophages and vascular smooth muscle cells engulfing large amounts of lipids and differentiate into foam cells, forming atherosclerotic plaques (30).

**Figure 7:**
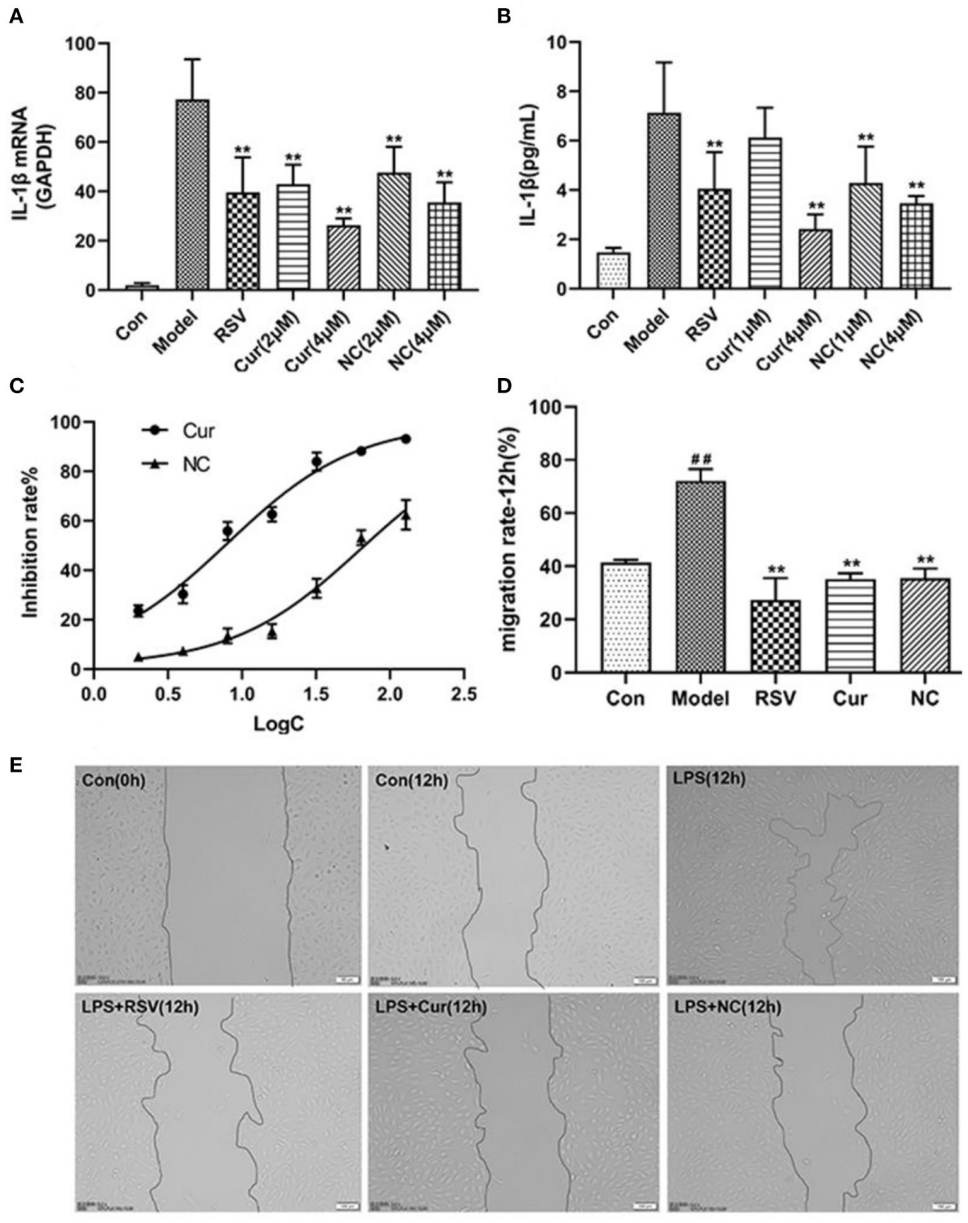
Effect of rosuvastatin (RSV), curcumin (Cur), and Nicotinic-curcumin (NC) on HUVECs in the presence of LPS. **(A)** ELISA test for the expression of IL-1β in HUVECs treatment with LPS, RSV, Cur, and NC; **(B)** RT-PCR results for the expression of IL-1β mRNA in HUVECs treatment with LPS, RSV, Cur, and NC; **(C)** The effect of different curcumin (Cur) and nicotinic-curcumin (NC) concentrations on proliferation of HUVECs. **(D)** Effect of LPS, RSV, Cur, and NC on migration of HUVECS. **(E)** Wound scratch assay for the migration of HUVECS after treatment with LPS, RSV, Cur, and NC (Data represents mean ± SD of 3 different experiments. *p < 0.05 and **p < 0.01, respectively). ^#^*p* < 0.05 and ^##^*p* < 0.01, respectively. ^#^: Compared with Con, *: compared with Model.

**Figure 8:**
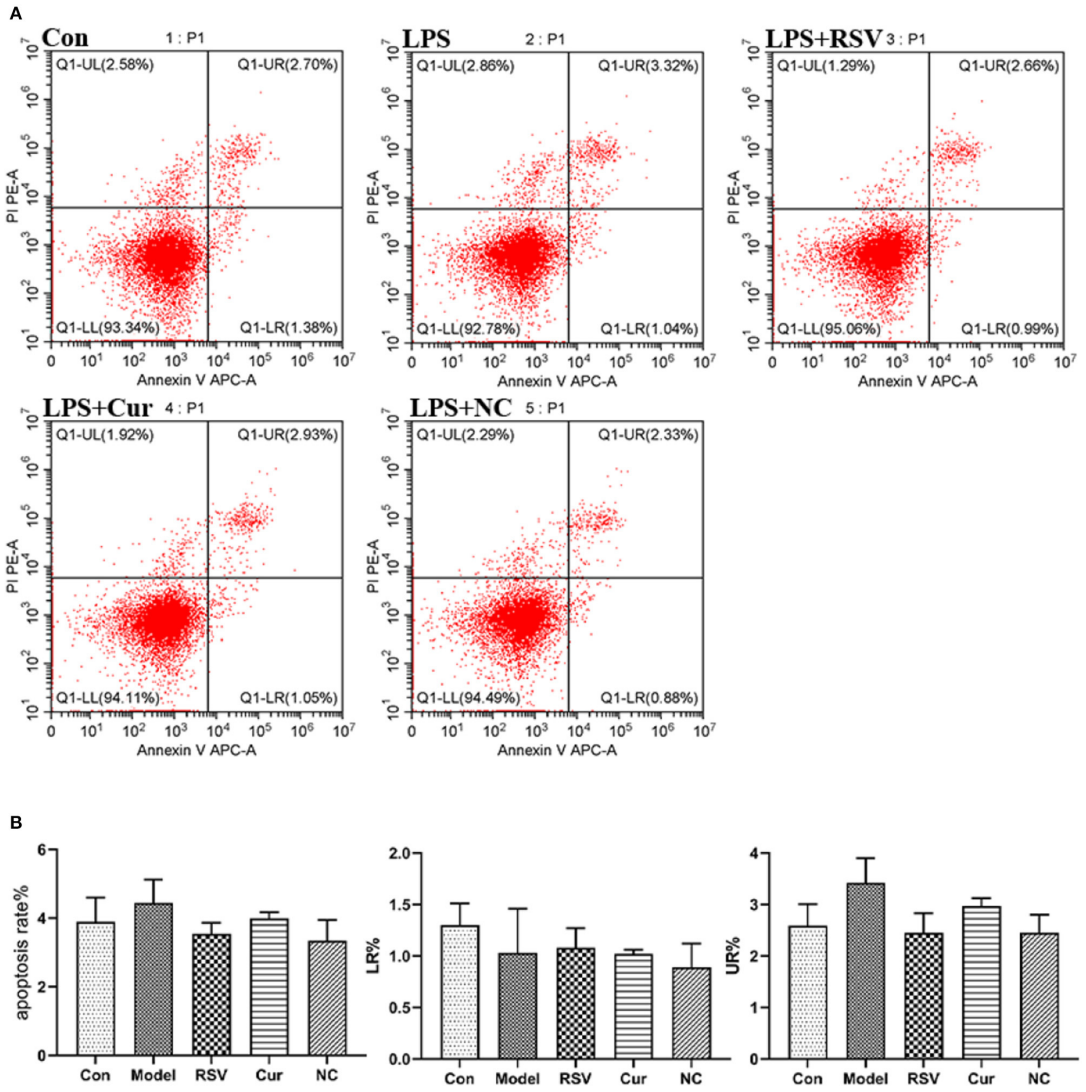
**(A)** Effect of LPS, RSV, Cur, and NC on apoptosis of HUVECS (apoptosis rate as a %, early apoptotosis (LR%) and late apoptotosis (UR%). **(B)** Degree of apoptosis on HUVECS induced by LPS, RSV, Cur, and NC.

ELISA and RT-PCR tests revealed that LPS simulation up-regulated secretion of IL-1β (*P* < 0.01; Figures 7A,B). However, NC treatment reversed this phenomenon. Comparatively, NC treatment (4 μM) exerted better anti-inflammatory effect than rosuvastatin (1 μM) (Figure 7). In addition, curcumin and NC significantly inhibited proliferation of HUVECs (P <0.01; Figure 7C). Wound scratch assay further revealed that LPS remarkably promoted the migration of HUVECs (*P* < 0.01). However, NC (4 μM) treatment inhibited the migration of HUVECs (*P* < 0.01; Figures 7D,E). Also, LPS and Cur exerted comparable apoptotic effect on HUVECS (Figures 8A,B).

### LPS Promotes the Release of HUVECs-Derived EVs but Curcumin and Nicotinic-Curcumin Inhibits This Effect

**Figure 9:**
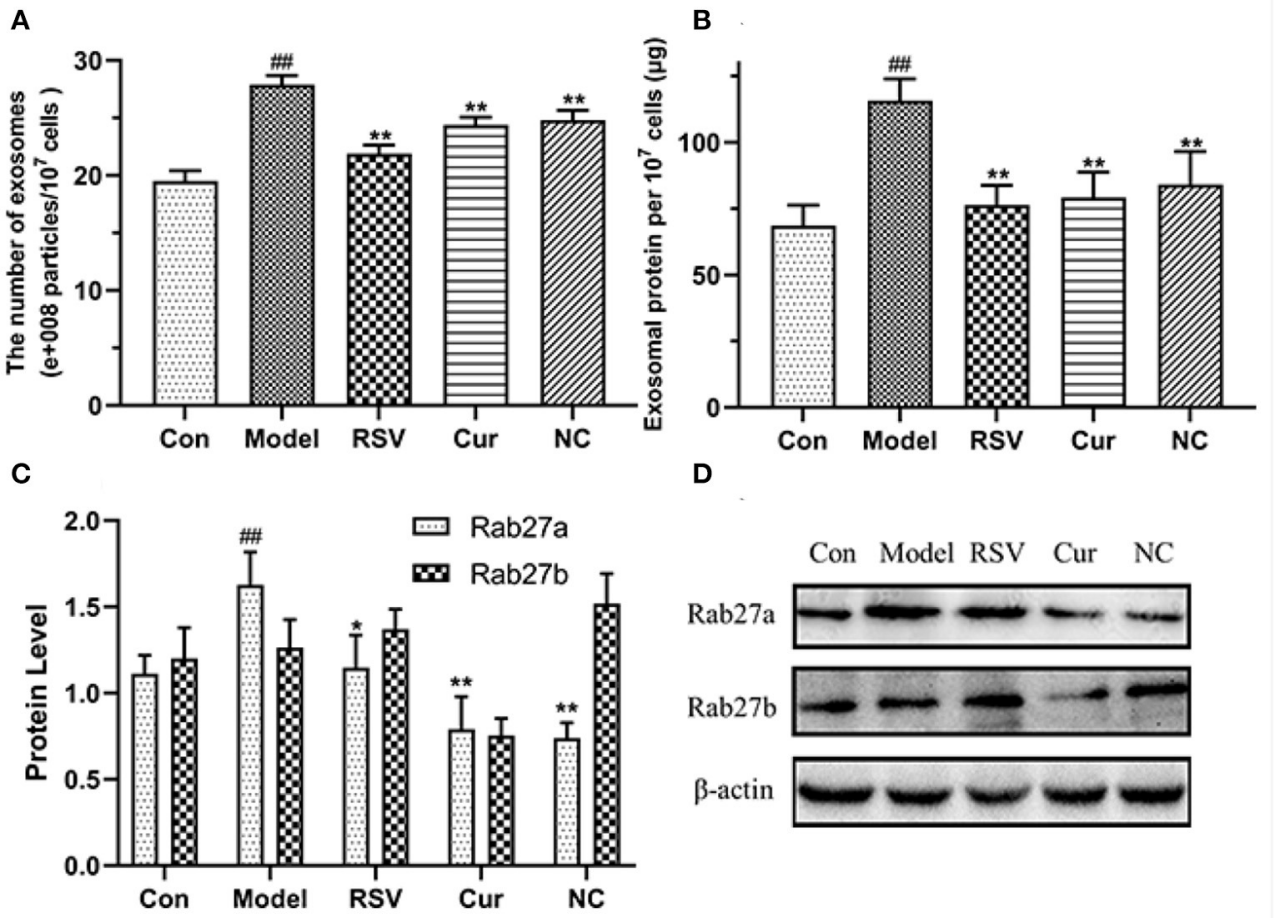
Effects of rosuvastatin (RSV), curcumin (Cur), and nicotinic-curcumin (NC) on production of EVs and inflammatory responses by HUVECs in the presence of LPS (1 μg/mL). **(A)** NTA for the number and size of EVs (30–150 nm); **(B)** BCA for the total concentration of EVs proteins; **(C,D)** Western blotting assay for the expression of Rab27a and Rab27b **(D)** proteins and the Rab27a and Rab27b levels were quantified **(C)**. ^#^*p* < 0.05 and ^##^*p* < 0.01, respectively. *p < 0.05 and **p < 0.01, respectively). **(C,D)** Western blotting assay for the expression of Rab27a and Rab27b(D) proteins and the Rab27a and Rab27b levels were quantified(C). ^#^: Compared with Con, *: compared with Model.

The total exosomal proteins content in each group was measured using the BCA method. The size and the concentration of the particles were also determined. Nanoparticle tracking analysis (NTA) and BCA analyses revealed that LPS treatment upregulated secretion of endothelial EVs. However, after curcumin (4 μM) and NC (4 μM) treatments significantly modulated production of EVs and inhibited inflammatory responses by HUVECs (Figures 9A,B). Western blot further revealed that LPS treatment up-regulated the expression of Rab27a. However, NC treatment downregulated expression of Rab27a but increased that of Rab27b (Figures 9C,D). Overall, NC reduced the secretion of EVs related to the inhibited expression of Rab27a proteins.

### The Effects of Curcumin and Nicotinic-Curcumin on the Expression of miR-125a-3p, miR-92a-3p, miR-126-5p, and miR-143-3p in Endothelial EVs

**Figure 10:**
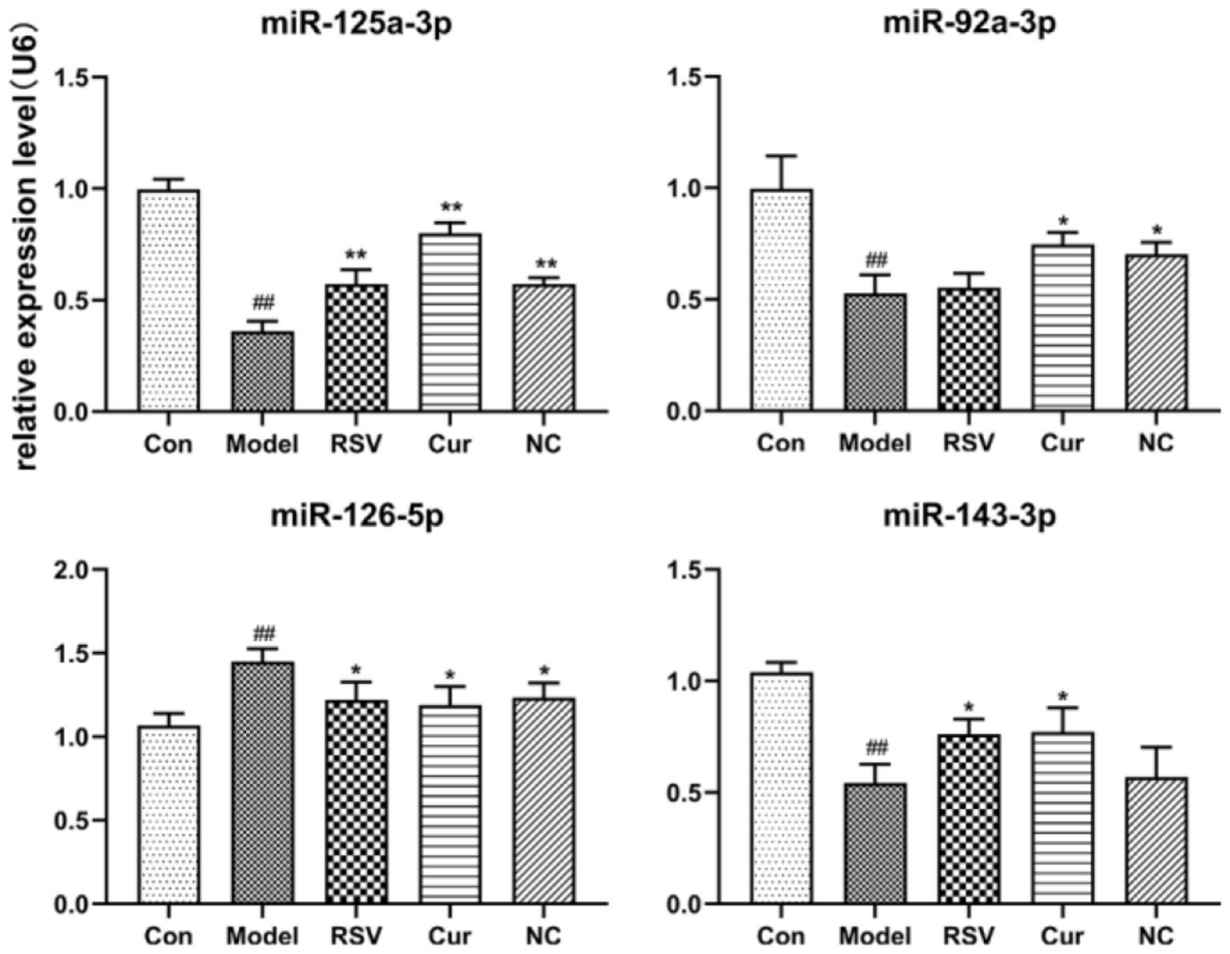
Effect of rosuvastatin (RSV), curcumin (Cur) and nicotinic-curcumin (NC) on the secretion of various miRNAs by HUVECs, in the presence of LPS (1 μg/mL). The relative expression of miR-92a-3p, miR-126-5p, miR-125a-3p, and miR-143-3p in HUVECs were analyzed using RT-PCR (The data represents mean ± SD of 6 independent analyses. ^#^*p* < 0.05 and ^##^*p* < 0.01, respectively. *p < 0.05 and **p < 0.01, respectively).

RT-PCR revealed that LPS treatment reduced the relative expressions of miR-125a-3p, miR-92a-3p, and miR-143-3p, but increased that of miR-126-5p. However, NC treatment up-regulated the relative expressions of miR-125a-3p and miR-92a-3p but down-regulated that of miR-126-5p. Interestingly NC treatment had no effect on that of miR-143-3p (Figure 10). Curcumin treatment only decreased the expression of miR-126-5p, but increased the expression of miR-125a-3p, miR-92a-3p, and miR-143-3p.

## Discussion

Communication between endothelial cells and vascular smooth muscle cells are essential in the progression of cardiovascular disease (20). We focused on regulatory effects of endothelial EVs from LPS activated or non-activated HUVECS on VSMCs. In general, EVs of non-activated HUVECS modulate AS by regulating proliferation, migration and lipid storage in VSMCs. However, LPS-induced EVs disrupt the beneficial functions of endothelial EVs from non-activated HUVECS. Further analyses revealed that LPS treatment dysregulated the expression of numerous miRNAs in endothelial EVs. The miRNAs participates in regulating several processess and aspects of vascular smooth muscle cells. LPS treatment regulates production of microRNAs in endothelial EVs. The role of miR-27a-3p is controversial. For instance, miR-27a-3p is thought to suppress expression of ARHGEF26 and inhibits proliferation of SMCs by interacting with a conserved binding site in the 3' untranslated region of ARHGEF26 mRNA (31). The 3' region contains multiple conserved binding sites for miR-27a-3p, which regulates the expression TSP-1 gene. This suggests that miR-27a-3p accelerates arteriosclerosis development by inhibiting the expression of mRNA for TSP-1 gene (31). Meanwhile, miR-27a-3p regulates endothelial inflammatory response by targeting MAPK, TGFBR1 and TGFBR2 signaling pathways (32). In coronary atherosclerosis, miR-365b-3p inhibits proliferation and migration of human coronary artery smooth muscle cells by directly targeting ADAMTS1 (33). Surprisingly, in our study, LPS treatment increased the expression of miR-365b-3p in endothelial EVs, which was contrary to the effect of LPS-induced EVs. This might be attributed to under-expression of miR-365b-3p, only a minor regulatory miRNA. MiR-126-5p modulates atherosclerosis, regulates endothelial stress response and promotes endothelial repair (34–36). At sites where disturbed laminar flow compromises endothelial cell (EC) function, atherosclerosis plaques developed preferentially is correlated with low miR-126-5p levels in endothelial cells. Endothelial miR-126-5p promotes endothelial proliferation and limits atherosclerosis by suppressing expression of delta-like 1 homolog (Dlk1) (37). It also promotes contractile switching of aortic smooth muscle cells by targeting VEPH1 and alleviates Ang II-induced abdominal aortic aneurysm in mice. MicroRNA-155-5p promotes progression of atherosclerosis (38) mainly by enhancing inflammatory response during the development of the disease by targeting SOCS1 via the STAT3 and NF-κB signaling pathways. MiR-155-5p also promotes cholesterol uptake on macrophages, which may be related to the accumulation of cholesterol in VSMCs (24, 39).

In ECs, let-7b-5p promotes angiogenesis by inhibiting TGFBR1, its target gene. In addition, let-7b-5p modulated the secretion of TNF and IL-6 by monocytes as well as expression of SERPINE1 in LPS-activated macrophage (40). Let-7b-5p also promotes the degradation of fatty acids in macrophages through mitochondrial respiration. The effect of let-7b-5p on the functioning of vascular smooth muscle cells is rarely reported. MiR-92a-3p promotes apoptosis of endothelial cells by targeting SIRT6 and activating MAPK signaling pathway (41). However, separate studies show that inhibition of miR-92a-3p regulates the development of atherosclerotic plaques (42, 43). Furthermore atheroprotective flow patterns decrease the level of miR-92a, which in turn increases KLF2 expression to maintain endothelial homeostasis (44). Up regulated expression of miR-30a-5p in atherosclerotic plaques suggest the miRNA is a potential diagnostic marker for atherosclerotic plaques. However, the regulatory property of miR-30a-5p needs further investigation (45). Interventional therapy in restenotic arteries of lower extremity substantial inhibits the expression of miR-125a-3p, relative to normal arteries. A more in-depth study revealed that miR-125a-3p effectively inhibits the proliferation and migration of VSMCs and the occurrence of vascular stenosis by targeting MAPK1 (23). Moreover, miR-125a-3p was over-expressed in ECs. Meanwhile, a separate study revealed that by inhibits the expression of vascular endothelial growth factor (VEGF), miR-125a-3p repressed angiogenesis in renal cancer tissues (46). LPS treatment mediated reduction of miR-125a-3p expression disrupted the normal functions of EVs. We found MiR-181a-2-3p possibly regulates the TLR/NFκB signaling and activates inflammatory and inflammasome responses. MiR-143-3p modulates both cellular and EVs-mediated responses in pulmonary vascular cells (47). Extracellular microvesicles containing miR-143-3p secreted by l-flow or KLF2 overexpressed in endothelial cells inhibits atherosclerosis in adjacent VSMCs (48). In addition, bidirectional movement of extracellular miR-143-3p may occur between SMCs and ECs under atherosclerotic conditions (49). Exosomal miR-21-3p from nicotine-treated macrophages increases migration and proliferation VSMC by targeting PTEN. However, the expression of the miRNA in ECs is very low (50). LPS down-regulated the expression of homologous miR-10a-5p and miR-10b-5p. The expression of miR-10a-5p negatively correlated to atherosclerosis progression. In macrophages, miR-10a-5p suppressed the expression of ligand-dependent nuclear receptor co-repressor, thereby promoting fatty acid oxidation (51). However, in a separate study, atherosclerotic plaques induced the over-expression of miR-10b-5p in VSMCs, which promoted the proliferation of these cells by targeting TIP30 via the Akt pathway (25).

Inflammation-induced dysregulated expression of miRNAs in EVs regulates pathogenesis of atherogenesis. Accordingly, such miRNAs are potential diagnostic markers for atherogenesis. Based on LPS-induced changes in the expression of miRNAs in endothelial EVs alone, we believe over-expression of miR-155-5p participates in accumulation of lipids in VSMCs. Also, over-expression of miR-125a-3p and miR-143-3p mediates the proliferation and migration of VSMCs induced by endothelial EVs. We also believe the LPS-induced proteomic changes in endothelial EVs are the major factor influencing the functioning of VSMCs.

Meanwhile, LPS treatment up-regulated the secretion of endothelial EVs. However, curcumin and nicotinic-curcumin treatment suppressed LPS-mediated over-production of IL-1β in endothelial cells during inflammation. In addition, curcumin and nicotinic-curcumin treatment reduced LPS-mediated secretion of EVs. Also, curcumin and nicotinic-curcumin treatment upregulated the expression of miR-125a-3p, miR-92a-3p, and miR-143-3p, but modulated that of miR-126-5p, suggesting the two treatments suppresses inflammation.

Summarily, endothelial cells can regulate functioning of vascular smooth muscle cells via EVs, thereby influencing progression of atherosclerosis. Inflammation dysregulates the expression of endothelial EVs, which may be related to the expression levels of several miRNAs in the EVs. Therefore, inhibition of endothelial inflammation can potentially modulate atherosclerosis. Drugs that regulate secretion and expression of miRNAs in endothelial EVs are potential treatments for atherosclerosis.

## Data Availability Statement

The raw data supporting the conclusions of this article will be made available by the authors, without undue reservation.

## Ethics Statement

The studies involving human participants were reviewed and approved by the Animal Ethics Committee of the Hunan University of Chinese Medicine (HN-LL-KY-2016–004–01). Written informed consent for participation was not required for this study in accordance with the national legislation and the institutional requirements.

## Author Contributions

DX and YL: data curation and formal analysis. DL: funding acquisition, project administration, resources, and supervision. DX and YQ: investigation. LZ and XLi: methodology. YC and XLin: software. DX and YH: writing—original draft. DX and DL: writing—review and editing. All authors contributed to the article and approved the submitted version.

## Conflict of Interest

The authors declare that the research was conducted in the absence of any commercial or financial relationships that could be construed as a potential conflict of interest.

## References

[1] Kobiyama K Ley K. Atherosclerosis. Circ Res. (2018) 123:1118–20. 10.1161/CIRCRESAHA.118.313816 30359201 PMC6298754

[2] Arnao V Tuttolomondo A Daidone M Pinto A. Lipoproteins in atherosclerosis process. Curr Med Chem. (2019) 26:1525–43. 10.2174/0929867326666190516103953 31096892

[3] Naoto K. Mechanism of development of atherosclerosis and cardiovascular disease in diabetes mellitus. J Atheroscler Thromb. (2018) 25:27–39. 10.5551/jat.RV17014 28966336 PMC5770221

[4] Petrie JR Guzik TJ Touyz RM. Diabetes, hypertension, and cardiovascular disease: clinical insights and vascular mechanisms. Can J Cardiol. (2017) 34:575–84. 10.1016/j.cjca.2017.12.005 29459239 PMC5953551

[5] Stankovic M Ljujic B Babic S Maravic-Stojkovic V Lukic ML. IL-33/IL-33R in various types of carotid artery atherosclerotic lesions. Cytokine. (2019) 120:242–50. 10.1016/j.cyto.2019.05.010 31132589

[6] Munjal A Khandia R. Atherosclerosis: orchestrating cells and biomolecules involved in its activation and inhibition. Adv Protein Chem Struct Biol. (2020) 120:85–122. 10.1016/bs.apcsb.2019.11.002 32085889

[7] Xu S Yin M Koroleva M Mastrangelo MA Zhang W Bai P. SIRT6 protects against endothelial dysfunction and atherosclerosis in mice. Aging. (2016) 8:1064–82. 10.18632/aging.100975 27249230 PMC4931854

[8] Kershaw KN Lane-Cordova AD Carnethon MR Tindle HA Kiang L. Chronic stress and endothelial dysfunction: the multi-ethnic study of atherosclerosis (MESA). Am J Hypertens. (2017) 30:75–80. 10.1093/ajh/hpw103 27585566 PMC5155567

[9] Sun H Krauss RM Chang JT Teng BB. PCSK9 deficiency reduces atherosclerosis, apolipoprotein B secretion, and endothelial dysfunction. J Lipid Res. (2018) 59:207–23. 10.1194/jlr.M078360 29180444 PMC5794417

[10] Siasos G Sara JD Zaromytidou M Park KH Coskun AU Lerman LO. Local low shear stress and endothelial dysfunction in patients with nonobstructive coronary atherosclerosis. J Am Coll Cardiol. (2018) 71:2092–102. 10.1016/j.jacc.2018.02.073 29747829

[11] Maguire EM Pearce SWA Qingzhong X. Foam cell formation: A new target for fighting atherosclerosis and cardiovascular disease. Vasc Pharmacol. (2018) 112:54–71. 10.1016/j.vph.2018.08.002 30115528

[12] Pegtel DM Gould SJ. Exosomes. Ann Rev Biochem. (2019) 88:487–514. 10.1146/annurev-biochem-013118-111902 31220978

[13] Tao SC Guo SC. Extracellular vesicles in bone: “dogrobbers” in the “eternal battle field”. Cell Commun Sign. (2019) 17:6. 10.1186/s12964-019-0319-5 30658653 PMC6339294

[14] Wang W Li Z Feng J. The potential role of exosomes in the diagnosis and therapy of ischemic diseases. Cytotherapy. (2018) 20:1204–19. 10.1016/j.jcyt.2018.06.012 30243925

[15] Wang H Lu Z Zhao X. Tumorigenesis, diagnosis, and therapeutic potential of exosomes in liver cancer. J Hematol Oncol. (2019) 12:133. 10.1186/s13045-019-0806-6 31815633 PMC6902437

[16] Fu M Gu J Jiang P Qian H Xu W Zhang X. Exosomes in gastric cancer: roles, mechanisms, and applications. Mol Cancer. (2019) 18:41. 10.1186/s12943-019-1001-7 30876419 PMC6419325

[17] Vázquez-Ríos AJ Molina-Crespo A Bouzo BL López-López R Moreno-Bueno G de la Fuente M. Exosome-mimetic nanoplatforms for targeted cancer drug delivery. J Nanobiotechnol. (2019) 17:85. 10.1186/s12951-019-0517-8 31319859 PMC6637649

[18] Colao IL Corteling R Bracewell D Wall I. Manufacturing exosomes: a promising therapeutic platform. Trends Mol Med. (2018) 24:242–56. 10.1016/j.molmed.2018.01.006 29449149

[19] Wu P Zhang B Shi H Qian H Xu W. MSC-exosome: a novel cell-free therapy for cutaneous regeneration. Cytotherapy. (2018) 20:291–301. 10.1016/j.jcyt.2017.11.002 29434006

[20] Li M Qian M Kyler K Xu J. Endothelial-Vascular smooth muscle cells interactions in atherosclerosis. Front Cardiovasc Med. (2018) 5:151. 10.3389/fcvm.2018.00151 30406116 PMC6207093

[21] Jia LX Zhang WM Li TT Liu Y Piao CM Ma YC. ER stress dependent microparticles derived from smooth muscle cells promote endothelial dysfunction during thoracic aortic aneurysm and dissection. Clin Sci. (2017) 131:1287–99. 10.1042/CS20170252 28468950 PMC5461939

[22] Jansen F Stumpf T Proebsting S Franklin BS Wenzel D Pfeifer P. Intercellular transfer of miR-126-3p by endothelial microparticles reduces vascular smooth muscle cell proliferation and limits neointima formation by inhibiting LRP6. J Mol Cell Cardiol. (2017) 104:43–52. 10.1016/j.yjmcc.2016.12.005 28143713

[23] Hu W Chang G Zhang M Li Y Yin L Huang Y. MicroRNA-125a-3p affects smooth muscle cell function in vascular stenosis. J Mol Cell Cardiol. (2019) 136:85–94. 10.1016/j.yjmcc.2019.08.014 31499051

[24] Yang Y Yang L Liang X Zhu G. MicroRNA-155 promotes atherosclerosis inflammation via targeting SOCS1. Cell Physiol Biochem. (2015) 36:1371–81. 10.1159/000430303 26159489

[25] Yu X Li Z Chen G Wu WK. MicroRNA-10b induces vascular muscle cell proliferation through Akt pathway by targeting TIP30. Curr Vasc Pharmacol. (2015) 13:679–86. 10.2174/1570161113666150123112751 25612666

[26] Adrian AL. The problem of curcumin and its bioavailability: could its gastrointestinal influence contribute to its overall health-enhancing effects? Adv Nutr. (2018) 9:41–50. 10.1093/advances/nmx011 29438458 PMC6333932

[27] Panahi Y Ahmadi Y Teymouri M Johnston TP Sahebkar A. Curcumin as a potential candidate for treating hyperlipidemia: A review of cellular and metabolic mechanisms. J Cell Physiol. (2017) 233:141–52. 10.1002/jcp.25756 28012169

[28] Gu HF Li HZ Tang YL Tang XQ Liao DF. Nicotinate-Curcumin impedes foam cell formation from THP-1 cells through restoring autophagy flux. PLoS ONE. (2016) 11:e0154820. 10.1371/journal.pone.0154820 27128486 PMC4851383

[29] Kun R Ting J Hui-Fang Z Yin L Guo-Jun Z. Apigenin retards atherogenesis by promoting ABCA1-Mediated cholesterol efflux and suppressing inflammation. Cell Physiol Biochem. (2018) 47:2170–84. 10.1159/000491528 29975943

[30] Gimbrone MA García-Cardeña G. Endothelial cell dysfunction and the pathobiology of atherosclerosis. Circ Res. (2016) 118:620–36. 10.1161/CIRCRESAHA.115.306301 26892962 PMC4762052

[31] Zahedi F Nazari-Jahantigh M Zhou Z Subramanian P Wei Y Grommes J. Dicer generates a regulatory microRNA network in smooth muscle cells that limits neointima formation during vascular repair. Cell Mol Life Sci. (2016) 74:359–72. 10.1007/s00018-016-2349-0 27622243 PMC11107738

[32] Zhou Q Gallagher R Ufret-Vincenty R Li X Olson EN Wang S. Regulation of angiogenesis and choroidal neovascularization by members of microRNA-23r~27~24 clusters. Proc Natl Acad Sci USA. (2011) 108:8287–92. 10.1073/pnas.1105254108 21536891 PMC3100947

[33] Qu Y Zhang N. miR-365b-3p inhibits the cell proliferation and migration of human coronary artery smooth muscle cells by directly targeting ADAMTS1 in coronary atherosclerosis. Exp Ther Med. (2018) 16:4239–45. 10.3892/etm.2018.6720 30402161 PMC6200964

[34] Weber C. MicroRNAs: from basic mechanisms to clinical application in cardiovascular medicine. Arterioscler Thromb Vasc Biol. (2013) 33:168–9. 10.1161/ATVBAHA.112.300920 23325472

[35] Boon RA Dimmeler S. MicroRNA-1^26^ in atherosclerosis. Arterioscler Thromb Vasc Biol. (2014) 34:e15–6. 10.1161/ATVBAHA.114.303572 24833799

[36] Leistner DM Boeckel JN Reis SM Thome CE De Rosa R Keller T. Transcoronary gradients of vascular miRNAs and coronary atherosclerotic plaque characteristics. Eur Heart J. (2016) 1738–49. 10.1093/eurheartj/ehw047 26916800

[37] Schober A Nazari-Jahantigh M Wei Y Bidzhekov K Weber C. MicroRNA-126-5p promotes endothelial proliferation and limits atherosclerosis by suppressing Dlk1. Nat Med. (2014) 20:368–70. 10.1038/nm.3487 24584117 PMC4398028

[38] Wei Y Nazari-Jahantigh M Chan L Zhu M Heyll K Corbalan-Campos J. The microRNA-342-5p fosters inflammatory macrophage activation through an Akt1- and microRNA-155-dependent pathway during atherosclerosis. Circulation. (2013) 127:1609–19. 10.1161/CIRCULATIONAHA.112.000736 23513069

[39] Nazarijahantigh MM Wei YY Noels HH Akhtar SS Zhe ZZ Koenen R. MicroRNA-155 promotes atherosclerosis by repressing Bcl6 in macrophages. J Clin Invest. (2012) 122:4190–202. 10.1172/JCI61716 23041630 PMC3484435

[40] Beltrami C Besnier M Shantikumar S Shearn AI Rajakaruna C Laftah A. Human pericardial fluid contains exosomes enriched with cardiovascular-expressed MicroRNAs and promotes therapeutic angiogenesis. Mol Ther. (2017) 25:679–93. 10.1016/j.ymthe.2016.12.022 28159509 PMC5363195

[41] Xu Y Miao C Cui J Bian X. miR-92a-3p promotes ox-LDL induced-apoptosis in HUVECs via targeting SIRT6 and activating MAPK signaling pathway. Braz J Med Biol Res. (2021) 54:e9386. 10.1590/1414-431x20209386 33470395 PMC7812905

[42] Wiese CB Zhong J Xu ZQ Zhang Y Solano MAR Zhu W Vickers KC. Dual inhibition of endothelial miR-92a-3p and miR-489-3p reduces renal injury-associated atherosclerosis. Atherosclerosis. (2019) 282:121–31. 10.1016/j.atherosclerosis.2019.01.023 30731284 PMC7484899

[43] Feinberg MW Moore KJ. MicroRNA regulation of atherosclerosis. Circ Res. (2016) 118:703–20. 10.1161/CIRCRESAHA.115.306300 26892968 PMC4762069

[44] Wu W Xiao H Laguna-Fernandez A Villarreal G Jr Wang KC Geary GG. Flow-Dependent regulation of kruppel-like factor 2 is mediated by MicroRNA-92a. Circulation. (2011) 124:633–41. 10.1161/CIRCULATIONAHA.110.005108 21768538 PMC3511909

[45] Vasuri F Ciavarella C Fittipaldi S Pini R Pasquinelli G. Different histological types of active intraplaque calcification underlie alternative miRNA-mRNA axes in carotid atherosclerotic disease. Virchows Arch. (2019) 476:307–16. 10.1007/s00428-019-02659-w 31506771

[46] Hou P Li H Yong H Chen F Bai J. PinX1 represses renal cancer angiogenesis via the mir-125a-3p/VEGF signaling pathway. Angiogenesis. (2019) 22:507–19. 10.1007/s10456-019-09675-z 31254127

[47] Deng L Blanco FJ Stevens H Lu R Caudrillier A Mcbride MW. MicroRNA-143 activation regulates smooth muscle and endothelial cell crosstalk in pulmonary arterial hypertension. Circ Res. (2015) 117:870–83 10.1161/CIRCRESAHA.115.306806 26311719 PMC4620852

[48] Hergenreider E Heydt S Tréguer K Boettger T Horrevoets A Zeiher AM. Atheroprotective communication between endothelial cells and smooth muscle cells through miRNAs. Nature Cell Biol. (2012) 14:249–56. 10.1038/ncb2441 22327366

[49] Climent M Quintavalle M Miragoli M Chen J Condorelli G Elia L. TGFβ triggers miR-143/145 transfer from smooth muscle cells to endothelial cells, thereby modulating vessel stabilization. Circ Res. (2015) 116:1753–64. 10.1161/CIRCRESAHA.116.305178 25801897

[50] Zhu J Liu B Wang Z Wang D Wang Y. Exosomes from nicotine-stimulated macrophages accelerate atherosclerosis through miR-21-3p/PTEN-mediated VSMC migration and proliferation. Theranostics. (2019) 9:6901–19. 10.7150/thno.37357 31660076 PMC6815950

[51] Wei Y Corbalán-Campos J Gurung R Natarelli L Zhu M Exner N. Dicer in macrophages prevents atherosclerosis by promoting mitochondrial oxidative metabolism. Circulation. (2018) 138:2007–20. 10.1161/CIRCULATIONAHA.117.031589 29748186

